# State-of-the-art surgery for sigmoid diverticulitis

**DOI:** 10.1007/s00423-021-02288-5

**Published:** 2021-09-23

**Authors:** Roberto Cirocchi, Paolo Sapienza, Gabriele Anania, Gian Andrea Binda, Stefano Avenia, Salomone di Saverio, Giovanni Domenico Tebala, Mauro Zago, Annibale Donini, Andrea Mingoli, Riccardo Nascimbeni

**Affiliations:** 1 Department of Medicine and Surgery, University of Perugia, 06100 Perugia, Italy; 2 Department of Surgery “Pietro Valdoni”, “Sapienza” University of Rome, 00161 Rome, Italy; 3 Department of Medical Science, University of Ferrara, 4121 Ferrara, Italy; 4 General Surgery, Biomedical Institute, 16157 Genoa, Italy; 5 University of Insubria, Varese, Italy; 6 Surgical Emergency Unit, John Radcliffe Hospital, Oxford University NHS Foundation Trust, Oxford, UK; 7 Department of Robotic and Emergency Surgery, Manzoni Hospital, 23900 Lecco, Italy; 8 Department of Molecular and Translational Medicine, University of Brescia, 25124 Brescia, Italy

**Keywords:** Diverticular disease, Acute diverticulitis, Management, Surgical treatment

## Abstract

**Background:**

In the last two decades, there has been a Copernican revolution in the decision-making for the treatment of Diverticular Disease.

**Purpose:**

This article provides a report on the state-of-the-art of surgery for sigmoid diverticulitis.

**Conclusion:**

Acute diverticulitis is the most common reason for colonic resection after cancer; in the last decade, the indication for surgical resection has become more and more infrequent also in emergency. Currently, emergency surgery is seldom indicated, mostly for severe abdominal infective complications. Nowadays, uncomplicated diverticulitis is the most frequent presentation of diverticular disease and it is usually approached with a conservative medical treatment. Non-Operative Management may be considered also for complicated diverticulitis with abdominal abscess. At present, there is consensus among experts that the hemodynamic response to the initial fluid resuscitation should guide the emergency surgical approach to patients with severe sepsis or septic shock. In hemodynamically stable patients, a laparoscopic approach is the first choice, and surgeons with advanced laparoscopic skills report advantages in terms of lower postoperative complication rates. At the moment, the so-called Hartmann’s procedure is only indicated in severe generalized peritonitis with metabolic derangement or in severely ill patients. Some authors suggested laparoscopic peritoneal lavage as a bridge to surgery or also as a definitive treatment without colonic resection in selected patients. In case of hemodynamic instability not responding to fluid resuscitation, an initial damage control surgery seems to be more attractive than a Hartmann’s procedure, and it is associated with a high rate of primary anastomosis.

## Quick reference/description

**Fig. 1:**
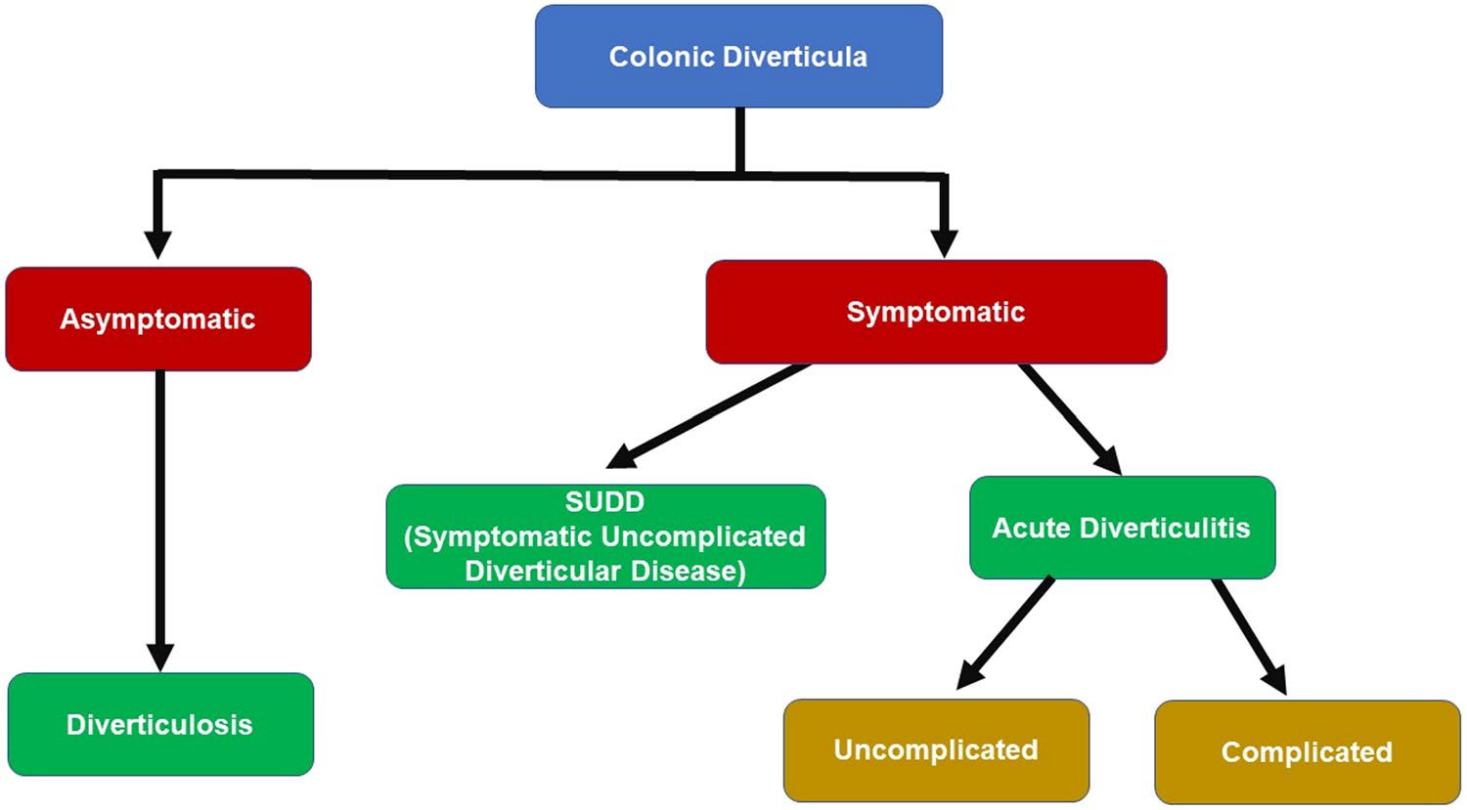
The natural history of diverticulitis

Sigmoid and left colonic diverticula are “pockets” of mucosa, submucosa, and serosa herniated from the bowel wall; in the western world, they present more often in the sigmoid, due to the intraluminal high pressure and the presence of weak spots in the muscular layer [1]. Colonic diverticulosis may be symptomatic; this heterogeneous condition is called Diverticular Disease (DD) and may be due to acute diverticulitis (AD) or persistent abdominal symptoms (abdominal pain, bloating, and changes in bowel habits) in the absence of macroscopic inflammation (SUDD = symptomatic uncomplicated diverticular disease). More often, it is an uncomplicated AD, but in some cases, an AD can become complicated with abscess, peritonitis, stricture, or hemorrhage (Fig. 1).

## Overview

In the Western population, DD is a quite common condition over the age of 50 (50% of the population). The sigmoid colon is the most frequent localization (65%), other uncommon locations being the sigmoid plus other parts of the large bowel (25%) or the entire colon (7%) [2]. Acute diverticulitis is the most common surgically treated disease after cancer; in the last three decades, the indications for surgical resection progressively reduced, in particular for the elective resection, and nowadays, elective surgery is considered only in the few cases with persistent symptoms and the risk of recurrent acute episodes in particular in young patients. Likewise, also, the indications for emergency surgery are quite restricted and limited to very selected cases (8.3%) [3] mostly for severe abdominal infective complications (41.62–79.4%) [4, 5].

## Anatomical considerations

The sigmoid is the last part of the colon and its morphology can be highly variable (length of S‐shaped loop between 11.9 and 91.1 cm, width of the sigmoid mesocolon between 4 and 11.5 cm) [6]. In common clinical practice, these variants may explain the heterogeneous clinical picture and the sometimes atypical presentation with right lower quadrant (RLQ) pain from sigmoid diverticulitis associated with a long and redundant colon [7].

## Indications for non-operative treatment or surgery

### Acute uncomplicated diverticulitis

The last few decades saw a revolution in the decision-making process for acute uncomplicated diverticulitis [1]. In the 1990s, guidelines suggested considering an elective colonic resection at the second clinical episode of severe diverticulitis [8], based on the high recurrence rate after the first episode (13.3%) and after the second episode (29.3%) [9]. This recommendation was based on the paper published by Parks in the 1960s, reporting data of 455 patients who had recurrent symptoms of acute diverticulitis in 7 to 45% of cases and only 6% of them responded to medical therapy after the third episode [10].

In the 1990s, the American Society of Colon and Rectal Surgery (ASCRS) (“Thus, after two attacks of uncomplicated diverticulitis, resection is recommended. Resection is also recommended for patients with complicated diverticulitis after one attack”) [11] and the European Association for Endoscopic Surgery (“Patients should be considered for elective surgery if they have had at least two attacks of symptomatic diverticular disease”) [12] supported the opinion of Parks, although it was not associated with robust scientific evidence [13]. In the same period, the Society for Surgery of the Alimentary Tract [14] categorized the indications for elective sigmoid resections: two or more severe acute attacks of diverticulitis despite successful medical treatment, single attack requiring hospitalization in a patient aged less than 40 years, one attack with evidence of contained perforation, colonic obstruction, or inflammatory involvement of the urinary tract and inability to rule out a colonic carcinoma.

Subsequently, other studies prompted the ASCRS to change the old paradigm to new statement, where the surgical option was decided case-by-case [15] (“The decision to recommend elective sigmoid colectomy after recovery from uncomplicated acute diverticulitis should be individualized”) [16, 17], effectively abandoning the number of attacks of colonic diverticulitis as a determinant of the surgical strategy [11]. In the ensuing years, other guidelines and statements supported this tailored approach (“Elective surgery to prevent complicated disease is not justified, irrespective of the number of previous attacks”… “The goal of elective surgery after one or more episodes of diverticulitis is to improve QoL. The indication should be individualized and based on the frequency of recurrences, duration, and severity of symptoms after the attacks and the comorbidity of the patient”) [18].

**Fig. 2:**
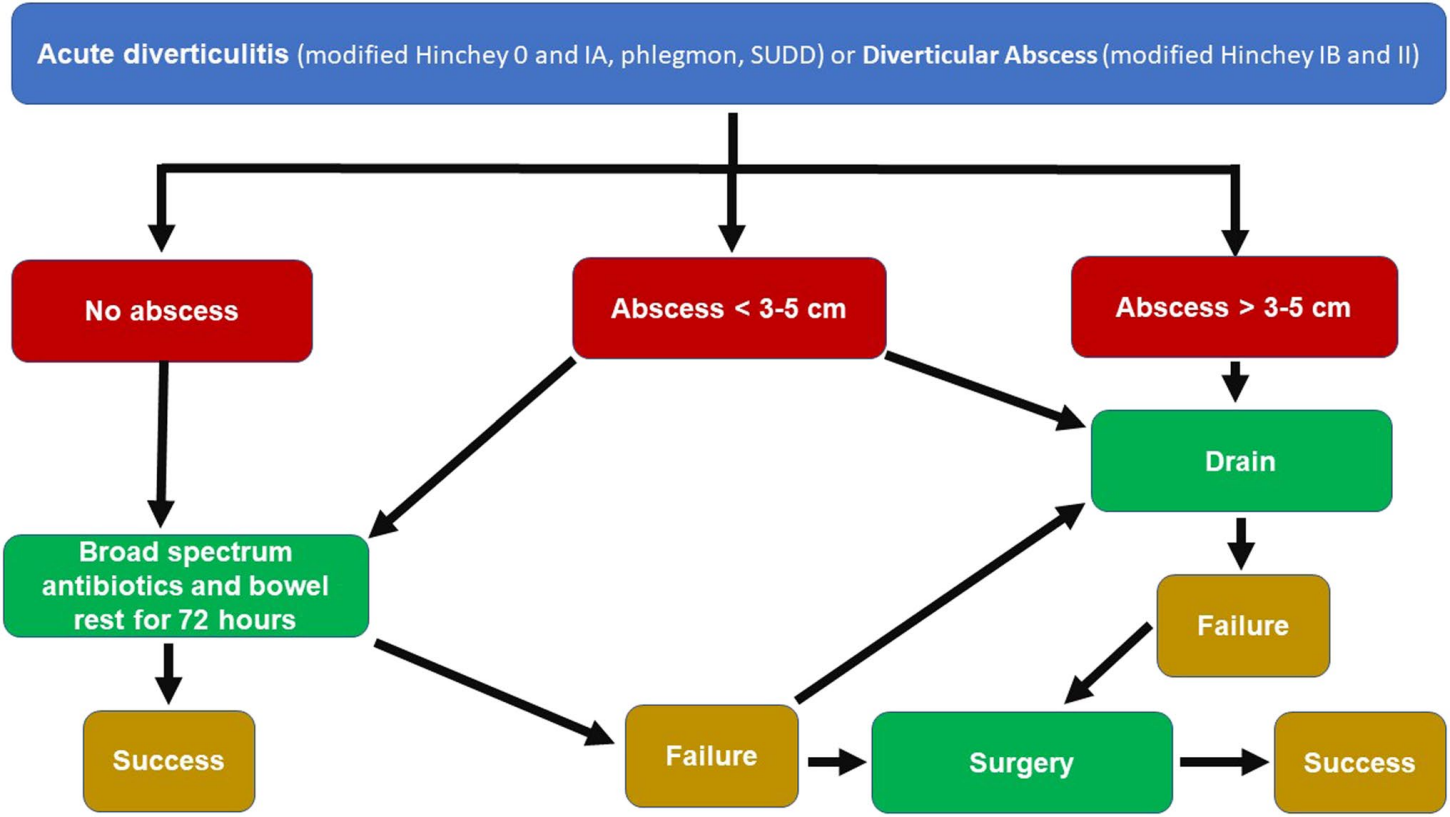
Treatment algorithm for patients with acute uncomplicated diverticulitis (modified Hinchey 0 and IA, phlegmon, SUDD) or diverticular abscess (modified Hinchey IB and II)

Nowadays, the uncomplicated diverticulitis (modified Hinchey 0 and IA, phlegmon, SUDD) is the most frequent diverticular condition, and it is commonly treated with antibiotics only, although recent studies demonstrated that in selected patients, antibiotics may not be necessary [19]. Nowadays, Non-Operative Treatment (NOT) is also considered for patients with complicated diverticulitis with abscess (modified Hinchey IB and II) [20], but this strategy is associated with a risk of failure (18.6%) [21] (Fig. 2).

ASCRS and ESCP guidelines suggest antibiotic therapy covering both Gram-negative and anaerobic bacteria [22]. This can be enough for small pericolic abscesses but should be associated to percutaneous drainage for large accessible pelvic abscesses [17, 18]. In the common clinical practice, an abscess is considered to be “large” if its diameter is more than 3–5 cm (image-guided percutaneous drainage is usually recommended for stable patients with abscesses > 3 cm in size) [17, 18] “We suggest to treat patients with large abscesses with percutaneous drainage combined with antibiotic treatment; whenever percutaneous drainage of the abscess is not feasible or not available, we suggest to initially treat patients with large abscesses with antibiotic therapy alone, clinical conditions permitting. Alternatively, operative intervention is required” [23, 24]. They are usually associated with a higher rate of recurrence [22].

During NOT, it is very important to set up a strict follow-up with repeated clinical evaluations, US, and laboratory analyses, to be able to spot the early signs of worsening clinical conditions, which may demonstrate the failure of the NOT. As suggested by Hanna and Kaiser, “within 72 h after initiation of appropriate treatment, symptoms, and objective parameters (pain, fever, leucocytosis, systemic inflammatory response syndrome (SIRS), etc.) must improve without exception or completely resolve/normalize” [25]. In the suspect of NOT failure, a prompt reply action is needed as previously reported by Hanna and Kaiser: “Repeat imaging to discern whether a drainable abscess has formed or a surgical intervention” [25].

In these patients, a surgical operation is needed only in case of “failure of percutaneous drainage and/or antibiotics, and in a critically ill or deteriorating patient” [26].

Although most of the acute diverticulitis cases (> 70%) are uncomplicated [25], the indications for elective surgical treatment in patients with acute uncomplicated diverticulitis are still object of controversy. Elective colonic resection has some advantages, including the very low risk of colonic perforation (5.5%) [27].

The DIRECT trial [28] reported an improvement of the Quality of Life (QoL) in patients who underwent surgery, and recently, the LASER trial [29] reported better results than conservative management. However, the Achille’s heel of elective surgery is represented by the risk of postoperative complications and the risk of recurrence of diverticulitis. In the DIRECT trial [28], 11% of surgically treated patients had anastomotic leak and 15% required reoperation at 5 years. The LASER trial reported lower rates of major post-operative complications (Clavien-Dindo grade III or higher in 5% of cases) and of recurrent diverticulitis within 6 months (31%).

Nowadays, on the basis of these results, the decision to consider an elective surgical resection in patients with recurrent uncomplicated diverticulitis is to be taken on a case-by-case basis considering frequency of recurrences, duration and severity of symptoms, postoperative QoL, immunosuppression, surgical risk score, and patient values [29–31].

In uncomplicated acute diverticulitis, the key point is to identify the patients who may benefit of an elective surgical resection and those who on the contrary present a high risk of surgical morbidity and mortality. The latter should be considered for a long-term medical treatment. In any case, the therapeutic choice must arise from a frank and informed discussion with the patients [22].

### Acute complicated diverticulitis

In patients with acute complicated diverticulitis, severe septic complications are the most common cause of emergency surgical treatment.

Acute generalized peritonitis (Hinchey III or IV) or abscess (Hinchey IIb) associated with sepsis are time-dependent emergencies, in which a delay of the treatment is inversely related with prognosis [33, 34]. The International Guidelines for Management of Sepsis and Septic Shock suggest, in fact, hemodynamic optimization and emergency surgical treatment as soon as possible [35]. Initial resuscitation is based on the hemodynamic response to fluid bolus [36] of 30 mL/kg [37] while previous evidence suggested a fixed bolous of crystalloids of 500 mL (fluid challenge) [38]. According to the mentioned guidelines, “fluid administration beyond initial resuscitation requires careful assessment of the likelihood that the patient remains fluid responsive” [35]. The use of vasoactive agents, such as norepinephrine, is mandatory in the absence of a significant compensative response to fluid resuscitation [39].

**Fig. 3:**
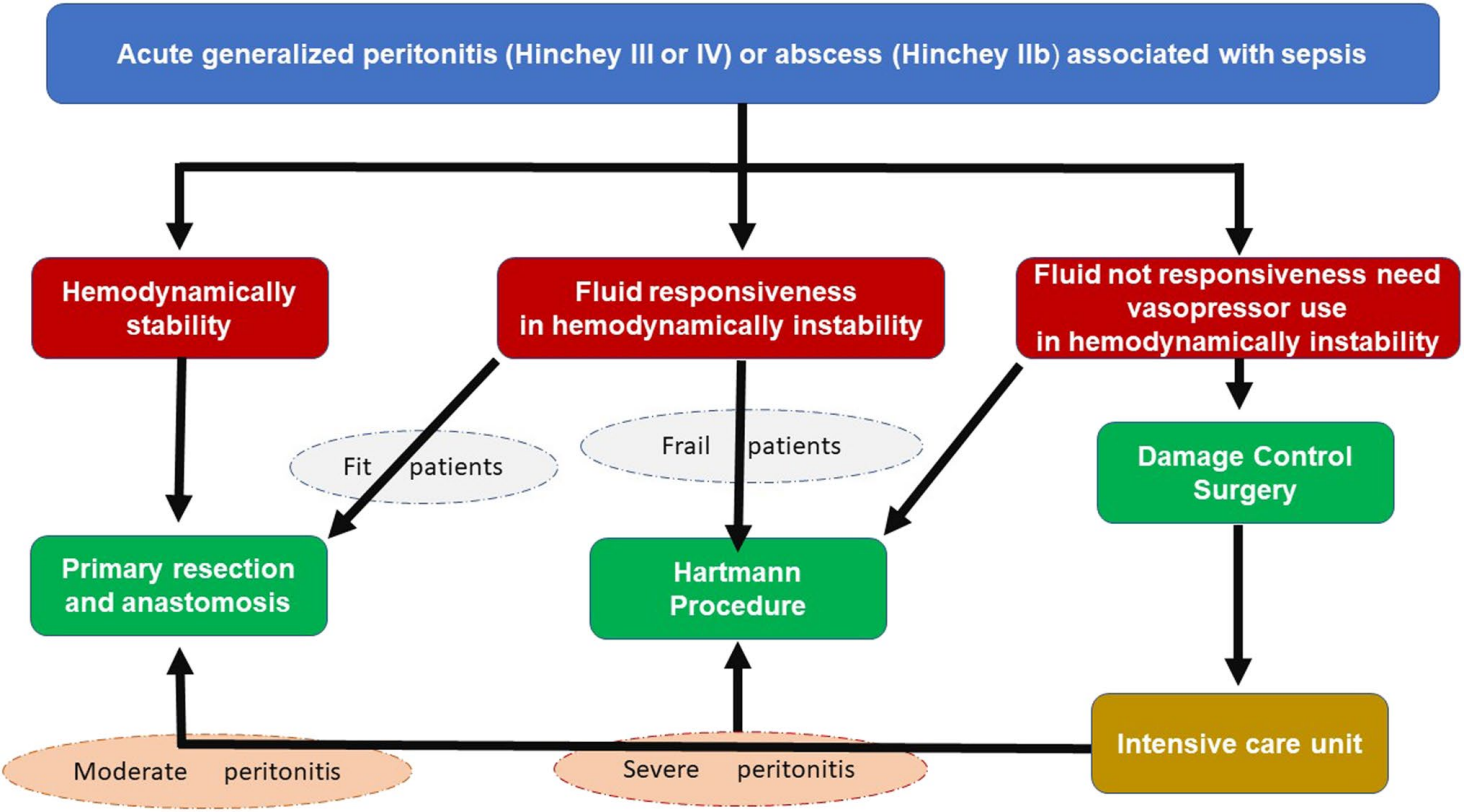
Treatment algorithm for patients with diverticular perforation and diffuse peritonitis (modified from Nascimbeni et al.: Management of perforated diverticulitis with generalized peritonitis. A multidisciplinary review and position paper) [40]

Nowadays, there is consensus [40, 41] on the fact that the emergency surgical approach to patients with sepsis and septic shock must be tailored on the basis of the hemodynamic conditions of the patient after an appropriate fluid resuscitation, as follows (Fig. 3):

#### Hemodynamically stable

**Fig. 4:**
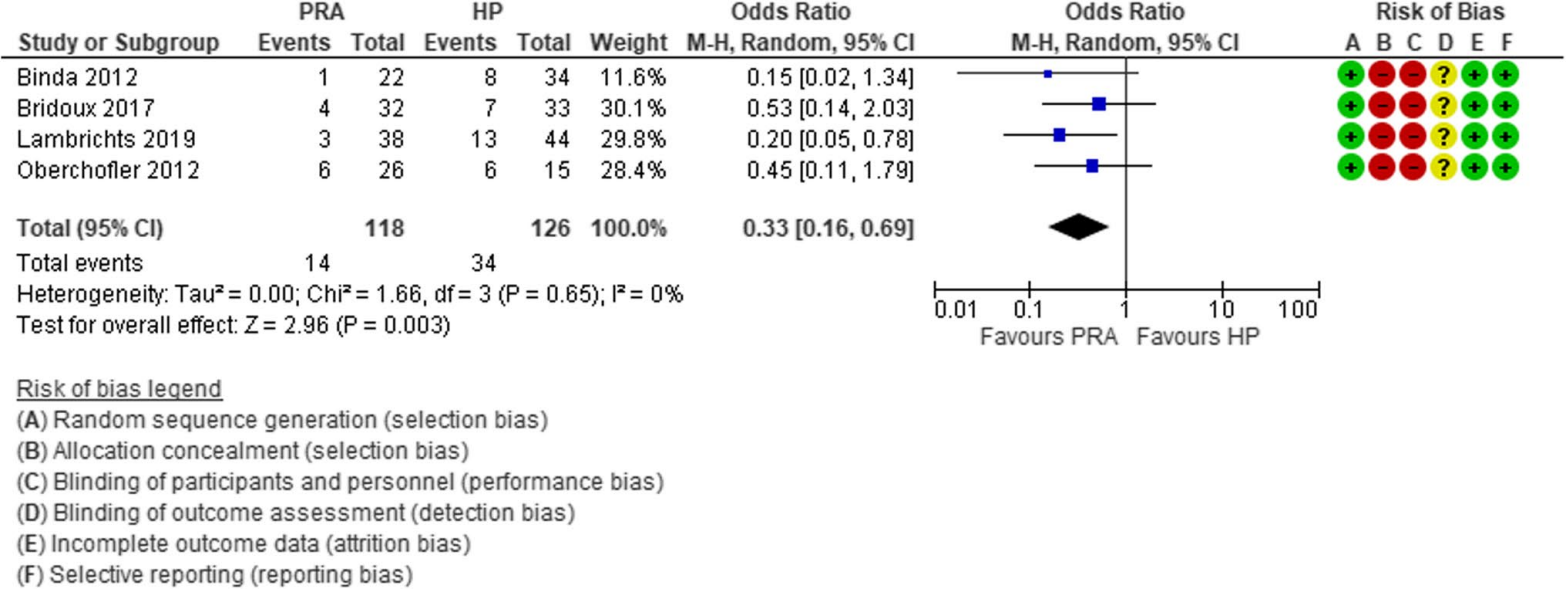
Forest plot of comparison: stoma-free patients who underwent sigmoid resection with primary anastomosis versus Hartmann’s procedure for perforated diverticulitis with purulent or fecal peritonitis in randomized controlled trials (modified from Lambrichts et al.: Sigmoid resection with primary anastomosis versus the Hartmann’s procedure for perforated diverticulitis with purulent or fecal peritonitis: a systematic review and meta-analysis) [48]

**Fig. 5:**
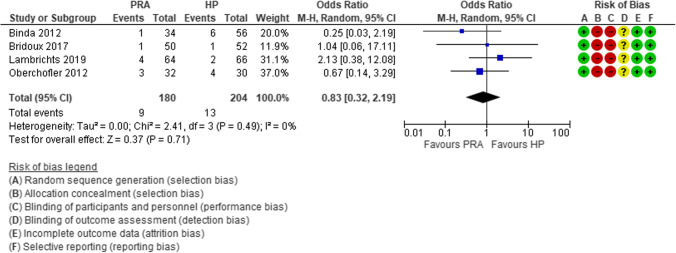
Forest plot of comparison: overall short-term mortality rates in patients who underwent sigmoid resection with primary anastomosis versus Hartmann’s procedure for perforated diverticulitis with purulent or fecal peritonitis in randomized controlled trials (modified from Lambrichts et al.: Sigmoid resection with primary anastomosis versus the Hartmann’s procedure for perforated diverticulitis with purulent or fecal peritonitis: a systematic review and meta-analysis) [48]

**Fig. 6:**
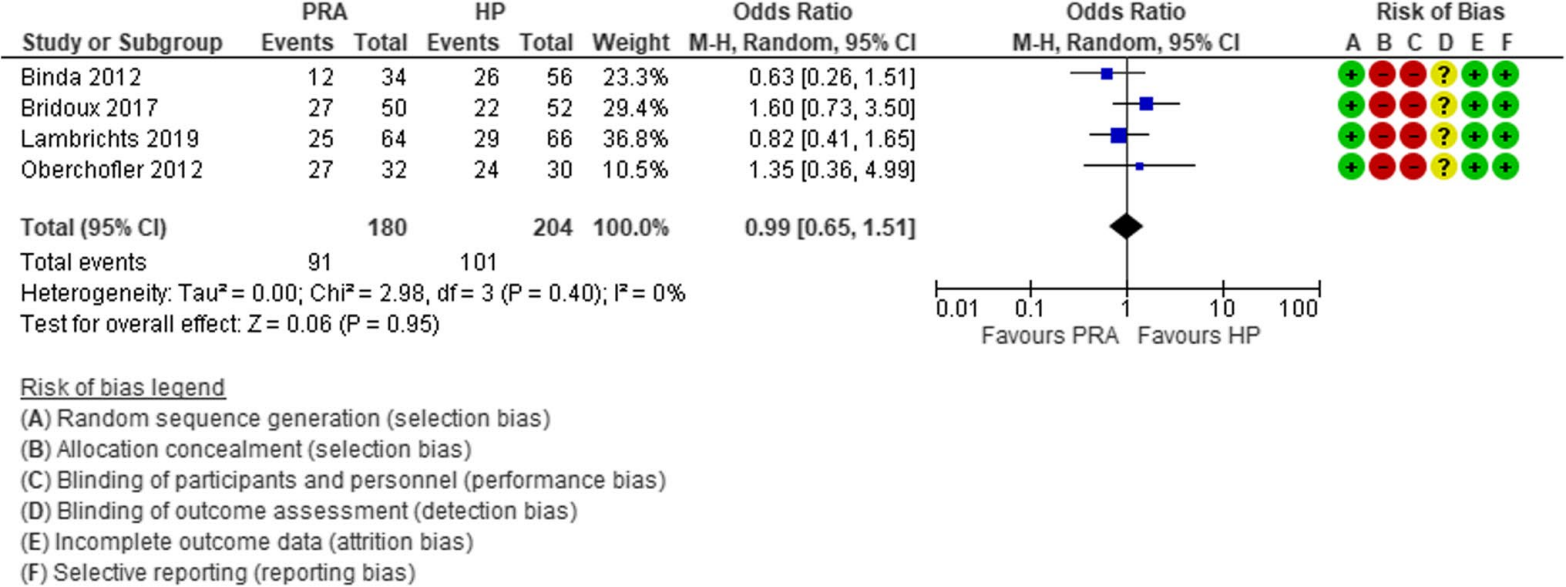
Forest plot of comparison: overall short-term morbidity rates in patients who underwent sigmoid resection with primary anastomosis versus Hartmann’s procedure for perforated diverticulitis with purulent or fecal peritonitis in randomized controlled trials (modified from Lambrichts et al.: Sigmoid resection with primary anastomosis versus the Hartmann’s procedure for perforated diverticulitis with purulent or fecal peritonitis: a systematic review and meta-analysis) [48]

For almost a century, the “ideal surgical treatment” for hemodynamically stable patients with acute complicated diverticulitis has been the Hartmann’s procedure (HP) [42]. More recently, some observational studies supported the immediate primary resection and anastomosis (PRA). Constantinides et al. performed a systematic review and meta-analysis clearly demonstrating the advantages of PRA with respect to the HP in terms of post-operative mortality, surgical site infections, abscesses, and peritonitis [43]. The main limitation of this review was the high risk of selection bias due to the retrospective nature of the included studies [43]. In the last decade, four RCTs were performed with the aim to compare PRA vs HP [44–47]. Lambrichts et al. reported the results of a meta-analysis of these four RCTs [48], showing that PRA is superior to HP as regards to the number of stoma-free patients (OR 0.33, 95% CI 0.16–0.69) (Fig. 4), stoma reversal rates (OR 2.62, 95% CI 1.29–5.31), and reversal-related morbidity (OR 0.33, 95% CI 0.16, 0.69), but there was no difference in short-term mortality (OR 0.83, 95% CI 0.32– 2.19) (Fig. 5), overall morbidity (OR 0.99, 95% CI 0.65, 1.51) (Fig. 6), and reintervention rates after the index procedure (OR 0.90, 95% CI 0.39–2.11).

Recent evidence suggests that the HP should only be indicated in severe generalized peritonitis or in critically ill patients [49], due to the high rate of permanent colostomy [50], poor quality of life [51, 52] and higher rate of post-operative complications associated with the Hartmann’s reversal [53]. In selected patients (i.e., those with severe local inflammation), PRA with diverting loop ileostomy can represent an alternative to HP with a reduced risk of anastomotic leak and related complications [26].

This new trend favoring the PRA is also supported by the clinical practice guideline recommendations recently published by the American Society of Colon and Rectal Surgeons (ASCRS) [17], the World Society Emergency Surgery (WSES) [23], the European Society of Coloproctology [18], and the European Association for Endoscopic Surgery/Society of American Gastrointestinal and Endoscopic Surgeons (EAES/SAGES) [54] (Table 1).

**Table 1:** Guidelines recommendations

Scientific societies	Reference	PRA vs HP	LL vs PRA or HP	DCS	Laparoscopy in elective surgery
ACRS	Hall 2020 [17]	The decision to restore bowel continuity should incorporate patient factors, intraoperative factors, and surgeon preference	Laparoscopic lavage is not recommended in patients with feculent peritonitis; rather, colectomy should typically be performed in this situation	NR	When expertise is available, a minimally invasive approachto colectomy for diverticulitis is preferred
EAES/SAGES	Francis 2019 [54]	In the appropriate clinical setting, we recommend consideration of sigmoid resection with primary anastomosis and proximal diversion over HP in patients with Hinchey III/IV diverticulitis	Laparoscopic lavage has been shown to decrease stoma formation rate without impacting 1-year mortality, although short-term morbidity may be increased. There was no consensus on an effective laparoscopic lavage technique	We recommend in unstable patients with perforated diverticulitis damage control strategies (resection without anastomosis, temporary abdominal closure, and second look) be considered	Laparoscopy is safe in the setting of elective surgery for diverticulitis and is associated with reduced rates of morbidity and length of stay compared to open surgery
ESCP	Schultz 2020 [18]	Primary anastomosis with or without diverting ileostomy can be performed in hemodynamically stable and immunocompetent patients with Hinchey III or IV diverticulitis	Laparoscopic lavage is feasible in selected patients with Hinchey III peritonitis. Alternatively, resection is recommended	There are some studies suggesting damage control with a second look within a couple of days	Elective colon resection for diverticulitis should preferably be performed laparoscopically when feasible
SICCR/ SICUT/ SIRM/ AIGO	Nascimbeni 2021 [40	Moreover, in stable patients with unfavorable risk assessment, laparoscopic HP might be an attractive option because the risk of incisional hernia is minimized and the adhesion formation is reduced, facilitating reversal	LPL may be effective in the management of purulent peritonitis reducing the rate of ostomy in selected patients. Its non-selective use results in high rates of unresolved sepsis and unplanned surgery	In the Transient Responder group, the temporary return of hemodynamic instability restricts surgical options to HP or to damage control surgery (DCS), which are chosen based on the evolution of the physiological derangement and secondarily on intra-abdominal severity assessment. In Not-Responder group, DCS is the most rationale immediate approach, due to the extreme exhaustion of the patient’s physiological reserves	NR
WSES	Sartelli 2020 [23]	We recommend Hartmann’s procedure for managing diffuse peritonitis in critically ill patients and in selected patients with multiple comorbidities	We suggest performing laparoscopic peritoneal lavage and drainage only in very selected patients with generalized peritonitis. It is not considered as the first line treatment in patients with peritonitis from acute colonic diverticulitis	We suggest damage control surgery (DCS) with staged laparotomies in selected unstable patients with diffuse peritonitis due to diverticular perforation	NR

ACRS, American Society of Colon and Rectal Surgeons; AIGO, Associazione Italiana Gastroenterologi Ospedalieri; EAES, European Association for Endoscopic Surgery; ESCP, European Society of Coloproctology; SAGES, Society of American Gastrointestinal and Endoscopic Surgeons; SICCR, Società Italiana di Chirurgia Colo-Rettale; SICUT, Società Italiana di Chirurgia d’Urgenza e del Trauma; SIRM, Società Italiana di Radiologia Medica; WSES, World Society Emergency Surgery; DCS, damage control surgery; HP, Hartmann’s procedure; LL, laparoscopic lavage; PRA, Primary resection and anastomosis

#### Responsive to fluids, hemodynamically unstable

In some septic patients with temporary hemodynamic instability returning to normal pressure after crystalloid infusion (at least 30 mL/kg or bolus of 500 mL), the best treatment is emergency resection of the perforated colonic segment and abdominal washout. The decision between a restorative (PRA) and non-restorative (HP) procedure is strictly linked to the physiological reserve associated with comorbidities and severity of generalized peritonitis. In elderly patients, the evaluation of comorbidities, as proposed by the Comprehensive Geriatric Assessment (CGA), represents a crucial point in the assessment [55]. The identification of the different comorbidity classes (fit, vulnerable, and frail) is extremely important: fit patients have a lower operative risk and are candidate to PRA; conversely, frail patients have a high operative risk [56] and need a HP [54].

#### Non-responsive to fluids and needing vasopressors, hemodynamically unstable

In case of hemodynamic instability unresponsive to fluid administration, damage control surgery (DCS) is more effective than the Hartmann’s procedure. DCS is a multistep strategy (abbreviated laparotomy, resuscitation in intensive care unit and definitive reconstruction) initially described for major trauma and lately applied to the surgical treatment of non-traumatic emergencies with severe sepsis or septic shock [57]. The first stage entails the local control of the septic source (peritoneal cleaning, limited resection of the perforated colonic segment with stapled off stumps) and Temporary Abdominal Closure (TAC); after 24–48 h of resuscitation in the ICU, a definitive surgical treatment is performed. In major trauma, the “lethal triad” (acidosis, hypothermia, and coagulopathy) creates a deadly cycle. Aim of DCS is to avoid very long extensive procedures in unstable patients undergoing resuscitation. DCS was proposed as a potential treatment for diffuse peritonitis due to perforated AD [58], but strategies and techniques have not been yet standardized [59] and the results are conflicting [60].

The recommendation to perform DCS in selected cases was first proposed in 2017 by the World Society of Emergency Surgery (WSES) Conference on the Management of Intra-abdominal infections [61], and successively was strongly supported by an update of the same surgical society (Consensus Conference 2020 update WSES [23]) and by the Consensus Conferences of EAES and SAGES [54].

DCS is associated with a reduction of post-operative mortality in patients poor general conditions (9.2% [95% CI 6.0 to 12.4%]). Another substantial advantage is the overall high rate of primary resection and anastomosis (62.1% [95% CI 40.8 to 83.3%]) [62, 63]. To date, criteria and techniques of DCS in the treatment of perforated diverticulitis with generalized peritonitis are yet to be defined [64].

## Surgical techniques

In the management of AD, another dilemma is the choice of the surgical access (i.e., conventional open or laparoscopic). Laparoscopic sigmoid resection is the preferred procedure in the elective setting. Since 1991, after the first report of laparoscopic colectomy [65], laparoscopic surgical access has gained a key role in the treatment of uncomplicated recurrent diverticular disease. This approach is commonly performed in the uncomplicated stages of acute diverticulitis with recurrent inflammatory episodes, but yet, diverticulitis complicated by complex abdominal abscesses with/without fistula (Hinchey IIb) does not represent an absolute contra-indication to the laparoscopic approach [66].

Multiple observational studies have reviewed the outcomes of laparoscopic sigmoid resection for uncomplicated diverticulitis [67–69]. A systematic review and meta-analysis of these non-randomised studies reported clear advantages of laparoscopy over open surgery in terms of significantly lower rate of overall morbidity [17% vs 27%; OR 0.46, 95% CI 0.25 to 0.84; 11 studies; *I*^2^ = 74%] and minor complications [9% vs 18%; OR 0.37, 95% CI 0.18 to 0.78; nine studies; *I*^2^ = 55%]. However, these data might be influenced by the selection bias of the retrospective or prospective cohort studies included into the analyses [70].

**Fig. 7:**
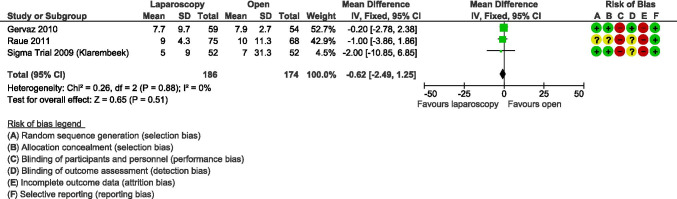
Forest plot of comparison: length of hospital stay in laparoscopic vs open resection for sigmoid diverticulitis (Modified from Abraha et al.: Laparoscopic versus open resection for sigmoid diverticulitis) [77]

**Fig. 8:**
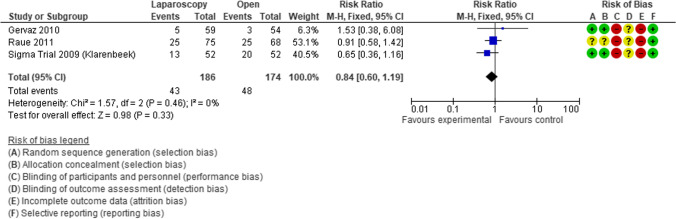
Forest plot of comparison: length of hospital stay in laparoscopic vs open resection for sigmoid diverticulitis (Modified from Abraha et al.: Laparoscopic versus open resection for sigmoid diverticulitis) [77]

Subsequently, Klarenbeek [71–73], Gervaz [74, 75], and Raue [76] presented the final results of three RCTs evaluating the benefits of elective laparoscopic vs open sigmoid resection for diverticulitis. In the Sigma trial, Klarenbeek showed that Quality of Life (QoL), measured by the Short Form-36 questionnaire 6 weeks after surgery, improved significantly after laparoscopic resection in terms of limitations due to physical health (PRF) (*p* = 0.039), emotional problems (*p* = 0.024), social functioning (*p* = 0.015), and pain (*p* = 0.032). On the other side, Gervaz and Raue did not report differences in QoL using the European Organization for Research and Treatment of Cancer Core Quality of Life Questionnaire (EORTC QLQ‐C30) v3 and the Gastrointestinal Quality of Life Index score, respectively. The meta-analysis of these three RCTs did not show any evidence to support the use of laparoscopic surgery in terms of a shorter length of stay (MD − 0.62, 95% CI − 2.49 to 1.25; *I*^2^ = 0%) (Fig. 7) or other postoperative outcomes including post-operative surgical complications (RR 0.84, 95% CI 0.60 to 1.19; *I*^2^ = 0%) (Fig. 8), 30-day postoperative mortality (RR 0.24, 95% CI 0.03 to 2.07; *I*^2^ = 0%), and operative time (MD 49.28 min, 95% CI 40.64 to 57.93; *I*^2^ = 0%). The meta-analysis on postoperative pain showed that laparoscopic surgery can reduce pain at postoperative day 4 (MD − 0.65, 95% CI − 1.04 to − 0.25; *I*^2^ = 0%) [77]. However, since the publication of these three RCTs, laparoscopic surgery greatly improved, and currently, many scientific societies support the safety and the advantages of the laparoscopic approach (Consensus Conference of EAES and SAGES [54], ESCP [18] and ASCRS [17]).

**Fig. 9:**
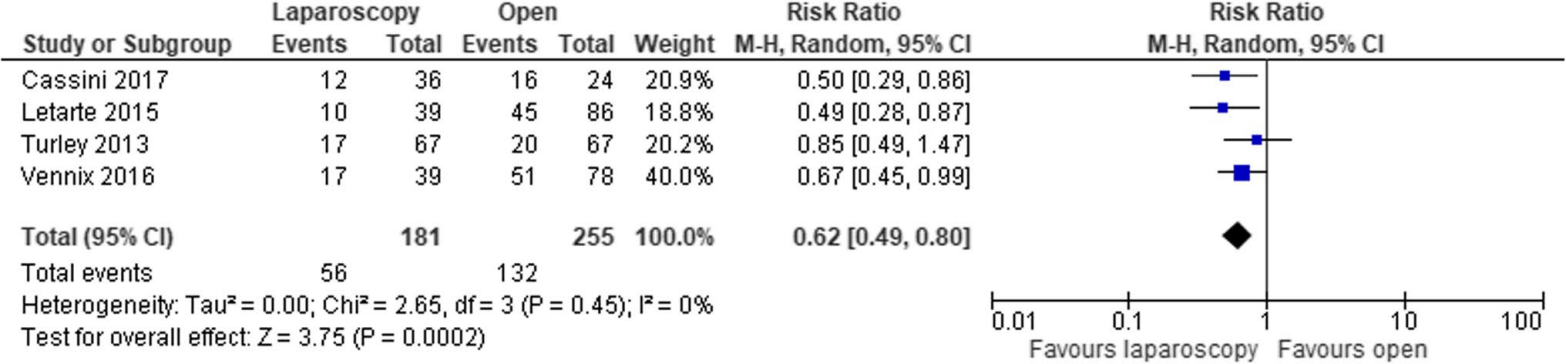
Forest plot of comparison: Post-operative complications in laparoscopic vs open sigmoidectomy in the emergency treatment of complicated sigmoid diverticulitis (Modified from Cirocchi et al: The role of emergency laparoscopic colectomy for complicated sigmoid diverticulitis: A systematic review and meta-analysis) [78]

**Fig. 10:**
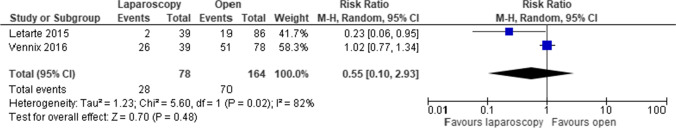
Forest plot of comparison: Rate of Hartmann procedure vs PRA after laparoscopic vs open sigmoidectomy (modified from Cirocchi et al.: The role of emergency laparoscopic colectomy for complicated sigmoid diverticulitis: A systematic review and meta-analysis) [78]

In the emergency setting and in hemodynamically stable patients, a laparoscopic approach is preferred, and surgeons with advanced laparoscopic skills reported some advantages in terms of lower postoperative complication rates; a systematic review and meta-analysis including 436 patients recruited from four observational studies highlighted that the laparoscopy resection slightly improves the rates of overall post-operative complications and post-operative length of stay, respectively (RR 0.62, 95% CI 0.49 to 0.80 and MD − 6.53, 95% CI − 16.05 to 2.99) (Fig. 9). However, this approach does not seem to affect other clinical outcomes (i.e., rate of Hartmann’s vs anastomosis, operating time, reoperation rate, and postoperative 30-day mortality) (Fig. 10) [78]. Furthermore, the Italian multisocietary position statement of Società Italiana di Chirurgia Colo-Rettale (SICCR), Società Italiana di Chirurgia d’Urgenza e del Trauma (SICUT), SIRM (Società Italiana di Radiologia Medica), and Associazione Italiana Gastroenterologi Ospedalieri (AIGO) recommended this approach if performed by qualified laparoscopic surgeons (In hemodynamically stable patients, confirmation and staging of diffuse peritonitis may be obtained by laparoscopy. In centers with adequate expertise selected cases may be handled by emergency laparoscopic procedures, either resective or non-resective) [40].

**Fig. 11:**
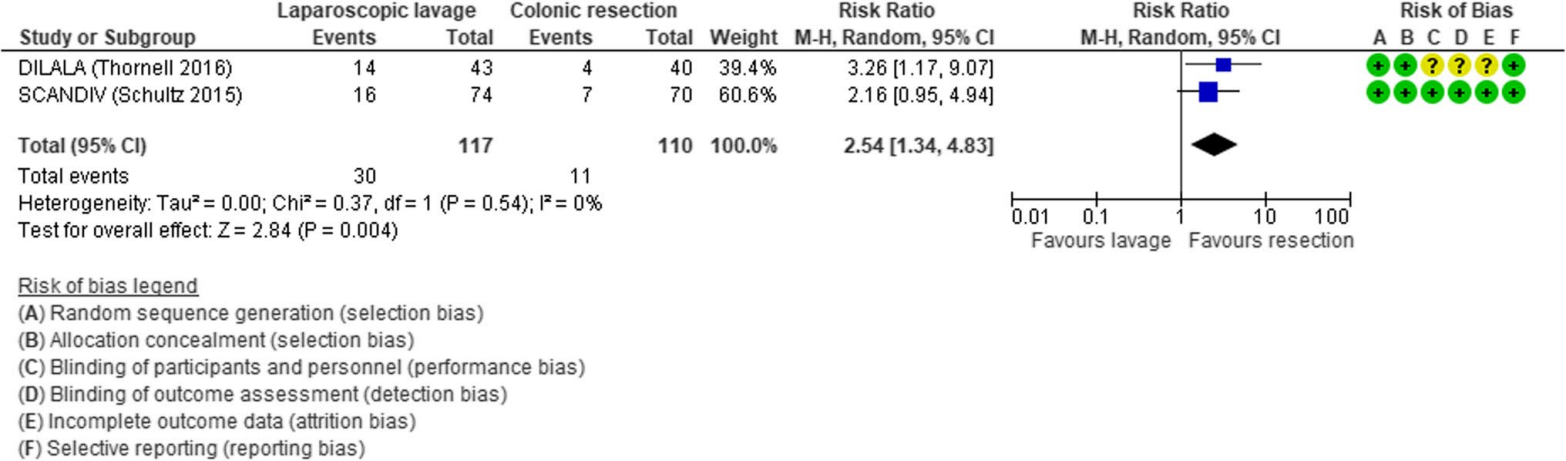
Forest plot of comparison: 90-day postoperative intra-abdominal abscess in laparoscopic lavage vs surgical resection for acute diverticulitis with generalized peritonitis (modified from Cirocchi et al.: Laparoscopic lavage versus surgical resection for acute diverticulitis with generalized peritonitis: a systematic review and meta-analysis) [80]

After the drainage of peritoneal collections, some surgeons advocated the use of laparoscopic lavage to improve the condition of septic patients through a dialytic reduction of endotoxin levels in the peritoneal fluid. The aim of the intraoperative laparoscopic lavage is the reduction of stoma rate and post-operative morbidity, as reported by the Consensus Conferences of EAES (European Association for Endoscopic Surgery) and SAGES (Society of American Gastrointestinal and Endoscopic Surgeons) [54]. This technique is feasible and safe, but advantages have been reported only in a selected group of patients, the success factors of LPL being still undefined [79]. In actual facts, a non-selective use of this technique causes a significant increase of postoperative intra-abdominal abscesses (RR 2.54, 95% CI 1.34–4.83) (Fig. 11) [80]. Different results reported in the three RCTs (LADIES) [81–83] (SCANDIV) [84, 85] (DILALA) [86, 87] may possibly be due to the use of different selection criteria. The most recent guidelines suggest laparoscopic peritoneal lavage only in selected patients with Hinchey III peritonitis (American Society of Colon and Rectal Surgeons [17], World Society Emergency Surgery [23] and European Society of Coloproctology [18]).

## Complications

Despite technical improvements and new surgical devices, anastomotic leak (AL) remains the most common cause of post-operative mortality and complications after sigmoidectomy [88]. Even if several studies identified several risk factors for AL (such as male sex, elderly age, obesity, severe comorbidities, prolonged surgery time, perioperative blood transfusions, and low anastomosis), an accurate anastomotic technique (tension-free, adequate blood supply, inverted anastomosis) is mandatory in order to reduce the incidence of AL [89]. Moreover, an anastomosis should be avoided in patients in critical conditions, under inotropes and on long-term steroid treatment.

## Pitfalls

**Fig. 12:**
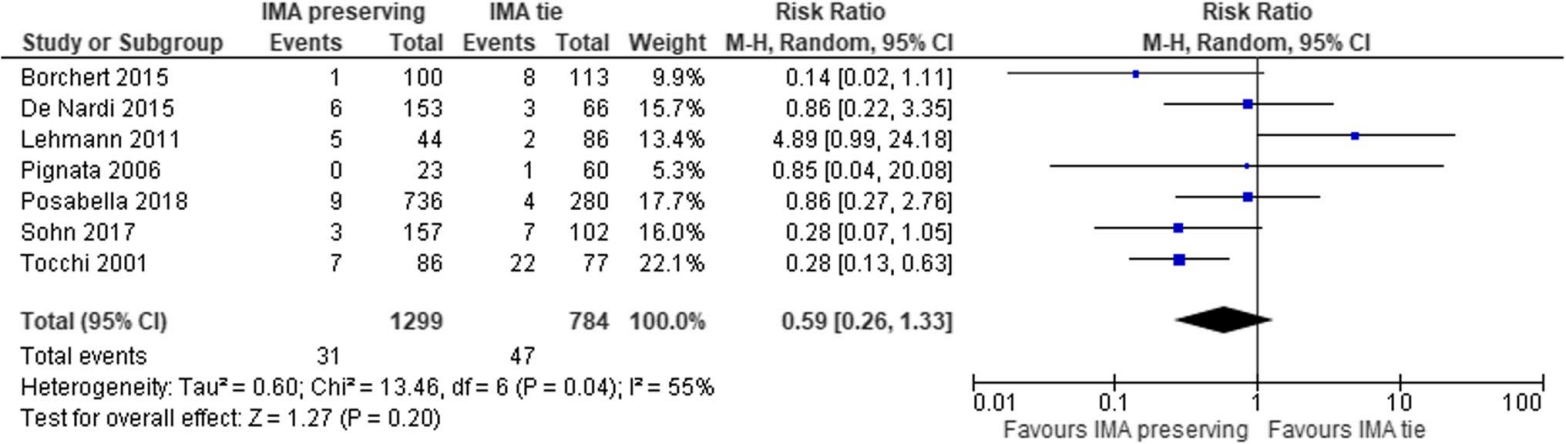
Forest plot of comparison: anastomotic leakage in IMA preserving group vs tie group (modified from Cirocchi et al.: Sigmoid resection for diverticular disease - to ligate or to preserve the inferior mesenteric artery? Results of a systematic review and meta-analysis) [90]

The ESCP recommended the preservation of IMA (In cases where there is no suspicion of cancer, IMA-preserving surgery can be performed to optimize preservation of the vascularization and the autonomic nerves) [18] although a meta-analysis failed to demonstrate the advantages of IMA preservation at reducing the risk of anastomotic leakage (RR 0.59, 95% CI 0.26–1.33) (Fig. 12) [90]. The IMA-preserving technique is performed by ligating the sigmoid vessels close to the colon, but this manoeuvre can be challenging due to mesosigmoid fibrosis commonly associated with the inflammation of the myoenteric plexus (40%) [91]. For this reason, the skeletonization of the sigmoid mesentery close to the bowel wall might be safer with the use of “cut and tie” devices [92]. Another option would be the much easier low ligation of the IMA after the origin of the left colic artery. With this manoeuvre, the IMA is prepared at its bifurcation, well away from the preaortic and hypogastric nervus plexa, where it can be easily ligated along with the IMV that runs exactly above the bifurcation. This approach looks much more prudent than the intramesosigmoid dissection also as it guarantees an adequate lymphadenectomy in the case of an unexpected carcinoma in the surgical specimen.

## Conclusions

In conclusion, the decision on whether to proceed with surgery in patients with DD should be individualized based on the most recent evidence. At present, judicious surgeons should exhaustively explain and discuss with the patients and their relatives about the uncertain benefits and the potential risks of the different type of surgical treatments and surgical access before performing the intervention in elective or emergency setting.

New studies are needed, especially in the long-term period, to provide additional data that can confirm the results of actually evidence reported.
